# Comparative genomics on chloroplasts of Chinese *Rubus*: genetic structure and phylogenetic relationships with other species of Rosaceae

**DOI:** 10.3389/fpls.2026.1765373

**Published:** 2026-03-20

**Authors:** Jian-Nan Chen, Chi-Zhong Feng, Dong-Xu Zhao, Le-Le Deng, Yuan-Ze Chen, Lu Jia, Hong-Xiao Ji, Mei-Xia Yang, Jing Li, Ti-Ran Huang, Lan-Qing Ma, Ming-Feng Yang

**Affiliations:** 1 College of Bioscience and Resources Environment, Beijing University of Agriculture, Beijing, China; 2 Key Laboratory for Northern Urban Agriculture of Ministry of Agriculture and Rural Affairs, Beijing University of Agriculture, Beijing, China; 3 Department of Criminal Science and Technology, Beijing Police College, Beijing, China

**Keywords:** chloroplast genome, phylogeny, Rosaceae, *Rubus*, structure and characteristics

## Abstract

As a large genus in Rosaceae, *Rubus* contains approximately 863 species worldwide, which distributed in the temperate regions of the Northern Hemisphere. The number of species in China accounts for approximately one quarter of the total, so China is the diversity distribution center of *Rubus* in the world. Given this, in this study, the chloroplast genomes and their characteristics of Chinese *Rubus* species were sequenced, assembled, and analyzed to provide molecular evidence for the *Rubus* systematic classification. The results indicated that the chloroplast genomes of *Rubus* exhibited a typical circular quadripartite structure, which consists of a large single-copy region (LSC), a small single-copy region (SSC), and a pair of inverted repeat regions (IR). Its size ranged from 155,472 to 156,886 base pairs (bp). It contained 131 genes, including 88 protein-coding genes (PCGs), 35 tRNA genes, and 8 rRNA genes. The *Rubus* chloroplast genome showed a high degree of conservation in gene arrangement, in the occurrence frequencies of different types of long repeat sequences, in simple sequence repeats (SSRs), in codon usage patterns, and in selection pressures on *PCG*s. Differences were mainly manifested in subtle expansion and contraction changes in the inverted repeat (IR) regions, as well as variations in the number of SSRs and long repeat sequences. In some branches, certain genes underwent enhanced or relaxed purifying selection. Phylogenetic analysis revealed that Chinese endemic *Rubus* species could be divided into eight evolutionary branches, which corresponding to the eight subgenera of *Rubus*. These data not only clarify the phylogenetic relationships among the Chinese Rubus species, but also provide the basic data for the taxonomic revision under the level of Chinese Rubus subgenera.

## 1 Introduction

The genus *Rubus* (Rosaceae) has been recognized as one of taxonomically challenging groups due to agamospermy (asexual seed production), apomixis, hybridization, and polyploidy (Weber, 1996; Howarth et al., 1997; Alice and Campbell, 1999; Michael, 2006). The species number of this genus ranged from 250 (Mabberley, 2017), 700 (Robertson, 1974; Lu and Boufford, 2003), 750 (Yu and Lu, 1985; Hummer, 1996), 600-800 (Thompson, 1995), 863 (Huang et al., 2023) to 1534 (Jennings, 1988; Royal Botanic Gardens, Kew, 2026). *Rubus* species are found worldwide except in Antarctica and are abundant in the Northern Hemisphere, with very few species occurring in the Southern Hemisphere (Focke, 1910, 1911, 1914;Yu and Lu, 1985; Yang et al., 2021). Focke (1910, 1911, 1914) established the first and most recent global (> 100 years old) infrageneric classification system of the genus, and recognized about 429 *Rubus* species in 12 sugenera, the three largest being *R*. subg. *Idaeobatus* (raspberries, 117 species), *R*. subg. *Malachobatus* (115 primarily Asian species) and *R*. subg. *Rubus* (blackberries, 132 species). Among the other nine subgenera (*R*. subg. *Anoplobatus*, *R*. subg. *Chamaebatus*, *R*. subg. *Chamaemorus*, *R*. subg. *Comaropsis*, *R*. subg. *Cylactis*, *R*. subg. *Dalibarda*, *R*. subg. *Dalibardastrum*, *R*. subg. *Lampobatus* and *R*. subg. *Orobatus*), only three have more than six species. In China, Yu and Lu (1985) accepted Focke’s section treatment of *Rubus*, instead of subgenus, and divided Chinese *Rubus* into 8 sections corresponding to Focke’s subgenera, the two largest sections are *R*. sect. *Idaeobatus* (88 species) and *R*. sect. *Malachobatus* (92 species). The species number of the other six sections rang from one (*R*. sect. *Chamaebatus*, *R*. sect. *Lampobatus*, and *R*. sect. *Rubus*) to eleven (*R*. sect. *Dalibardastrum*), in which, 139 species are endemic. Thus, China, especially south-western China is the modern distribution and differentiation center of *Rubus*, and is also an important region center of *Rubus* (Carter et al., 2019). Several attempts have been made to unravel the overall phylogeny within the genus based on ITS (Alice and Campbell, 1999; Alice et al., 2008; Wang et al., 2016; Yang and Pak, 2006) sequence data, exon sequence gene (Carter et al., 2019), and a few other nuclear and chloroplast loci, including *GBSSI-1* (Wang et al., 2019), *GBSSI-2* (Wang et al., 2016), *LEAFY* (Yang et al., 2012), *ndhF* (Howarth et al., 1997; Zhang et al., 2015), *PEPC* (Wang et al., 2016), *rbcL* (Wang et al., 2016), *rpl16* (Alice et al., 2008), *rpl20*-*rps12* (Wang et al., 2016), *trnL-trnF* (Alice et al., 2008; Yang and Pak, 2006), *trnG-trnS* (Michael, 2006; Wang et al., 2016), and chloroplast sequences (Carter et al., 2019). These studies proved that the genus *Rubus* was a monophyletic group, but of the subgenera, only *R*. subg. *Anoplobatus* and *R*. subg. *Orobatus* appeared to be monophyletic, the other recognized subgenera were para- or polyphyletic. Until 2023, Huang et al., revised this genus, and propose a new classification system with 10 subgenera: *R*. subg. *Anoplobatus*, *R*. subg. *Batothamnus*, *R*. subg. *Chamaerubus*, *R*. subg. *Cylactis*, *R*. subg. *Dalibarda*, *R*. subg. *Idaeobatus*, *R*. subg. *Lineati*, *R*. subg. *Malachobatus*, *R*. subg. *Melanobatus*, and *R*. subg. *Rubus*. For all this, the chloroplast genome structure and characteristic analysis of *Rubus* has not received sufficient attention, especially, species endemic to China are rarely involved. Thus, more comprehensive interpretation of the chloroplast genome structural characteristics and phylogenetic relationships among Chinese *Rubus* species is necessary.

Chloroplast is the most important and common plastid in plant cells and originates from endosymbiosis between independent living cyanobacteria and a nonphotosynthetic host with independent genomes and evolutionary routes (Li et al., 2021; Gao et al., 2019). It plays an essential role in energy conversion, photosynthesis, manufacturing of amino acids, fatty acids, chlorophyll, caroten, and other compounds (Abdullah et al., 2019; Daniell et al., 2016). Chloroplast genome, which is usually inherited maternally in angiosperm but paternally in some gymnosperms, is generally quadripartite, double stranded, and circular in most angiosperms (Daniell, 2007; Guo et al., 2025; Ji et al., 2025). It consists of one large single copy (LSC) region, two inverted repeats (IR) regions and one small single copy (SSC) region with genome of 75 kb to 292 kb, and includes *PCG*s, ribosomal RNA (rRNA), and transfer RNA (tRNA) genes. (Ahmed 2015; Huang et al., 2025; Li et al., 2025; Mo et al., 2025; Lee et al, 2025). However, linear chloroplast genome has also been reported in some species (Oldenburg and Bendich, 2004, 2015). Though genomic structure, gene content, gene order, and base composition of chloroplast are known to be highly conserved, especially in IR regions (Li et al., 2021; Lu et al., 2024), many mutational events also take place within chloroplast genome such as insertions and deletions (InDels), translocations, inversions, point mutations, single nucleotide polymorphism, and variations in the number of tandem repeats (Abdullah et al., 2019, 2025; Mehmood et al., 2020). These variations make chloroplast genome an ideal candidate for phylogenetic and population genetic studies and species identification at diverse taxonomic levels (Ahmed et al., 2020; Daniell et al., 2016; Liu et al., 2025; Skuza et al., 2025; Sun et al., 2021; Yang et al., 2019a, 2019b; Wang et al., 2025). With the advancement of high-throughput DNA sequencing technology, next-generation sequencing (NGS) has revolutionized plant phylogenetics, particularly through pan-plastome studies, by offering significantly enhanced resolution for reconstructing plant taxonomy and evolutionary relationships, or capturing inter- and intraspecific variations, or elucidating cultivation history of some ornamental, medicinal, and vegetable/food plant (Dong et al., 2025; Kan et al., 2024; Lin et al., 2026, 2025; Lu et al., 2023).

Here, we *de novo* sequenced and assembled the complete chloroplast genomes of forty-eight *Rubus* species (17 Chinese endemic *Rubus* species), and then compared the genomic structure and sequence variation of the chloroplast genome. We also performed comprehensive phylogenomic analyses to elucidate interspecific relationships, and identified loci with suitable polymorphism for *Rubus* species identification, and positive selection genes that potentially contributed to the adaptive evolution of Rosaceae species.

## 2 Materials and methods

### 2.1 Plant material, DNA extraction and sequencing

We selected 50 samples, including 48 *Rubus* species and 2 outgroups (*Filipendula palmata*, *Geum aleppicum*) (**Supplementary Table 1**). These samples were collected from the provinces of Heilongjiang, Xinjiang, Yunnan, Xizang, Sichuan, and Jiangxi. For the 19 newly sequenced samples, fresh leaves were dried and preserved in silica gel for DNA extraction, and the specimens were deposited in the Herbarium of Beijing Normal University (http://cls.bnu.edu.cn/). The leaves of *R*. *caesius* L., *R*. *chamaemorus* L., *R*. *gongshanensis* T.T. Yu & L.T. Lu, and *R*. *nyalamensis* T.T. Yu & L.T. Lu were obtained from specimens stored at the Herbarium of the Institute of Botany, Chinese Academy of Sciences (PE). Genomic DNA was isolated from silica-dried leaves, fresh leaves frozen at -80 °C, or herbarium specimens using the modified CTAB method (Doyle and Doyle, 1987). The concentration, integrity, and purity of total genomic DNA were assessed using an Agilent 5400. Qualified genomic DNA was fragmented using a Covaris ultrasonic disruptor, and the DNA library was constructed through end repair, A-tailing, adapter ligation, purification, and PCR amplification. The original image data files generated by high-throughput sequencing were converted into raw sequenced reads (raw data) via CASAVA base calling analysis and stored in FASTQ format (abbreviated as fq). Raw reads contain the sequence information of sequencing reads and their corresponding quality scores.

### 2.2 Assembly, annotation, and visualization of chloroplast genome

Raw reads were first filtered for quality scores below Q20 using CLC Genome Workbench v4.0.6 (QIAGEN, Hilden, Germany). Meanwhile, reads containing N bases and adapter sequences were removed from the raw data. GetOrganelle v1.7.6 (Jin et al., 2020) was used to assemble the complete chloroplast genome from the raw sequencing data. The online tool CPGAVAS2 (Shi et al., 2019) was employed for annotation. To further refine the annotation results, we referred to the complete chloroplast genome of seven species: *Arabidopsis thaliana* (OP474144), *R. buergeri* (NC_072261), *R. crassifolius* (NC_056941), *R. ichangensis* (NC_056935), *R. paniculatus* (PP566891), *R. rufus* (NC_056798), and *R*. *setchuenensis* (NC_056946). Collinearity analysis was executed meticulously by leveraging the MUMmer tool (Marçais et al., 2018). The gene arrangements of the chloroplast genomes among 48 *Rubus* species are generally conserved, with no significant abnormal breaks or irregular rearrangements, which are helpful for verifying the accuracy of genome assembly and annotation (**Supplementary Figure 1**). Then, we used the gbcheck sub-command of CPStools software (Huang et al., 2024) and the online tool GeSeq (Tillich et al., 2017) to verify the annotation accuracy of each gene in these plastid genomes. Subsequently, we manually corrected the annotation results of these chloroplast genomes, including those of special genes such as pseudogenes, trans-spliced genes, RNA-editing genes, and other genes that have not been correctly annotated, in order to obtain more accurate outcomes.

### 2.3 Border comparison and the characteristics analysis of the chloroplast genome

The chloroplast genome maps of Chinese *Rubus* species were generated using the online tool CPGview (Liu et al., 2023). The basic characteristics of these chloroplast genomes, including the length and GC content, the four main regions (LSC, IR, SSC, IRb), as well as the numbers of rRNA, tRNA, and CDS (coding sequences) were analyzed. Additionally, the relevant information of introns was obtained using Python scripts. These scripts are now also part of CGAS (Chloroplast Genome Analysis Suite), which is a comprehensive toolkit for chloroplast genome assembly, annotations, and comparative genomics (Abdullah et al., 2025). The CGAS repository is available at: https://github.com/abdullah30/Chloroplast-Genome-Analysis-Suite-CGAS.

In order to compare the contraction and expansion of the boundaries of the inverted repeat (IR) regions, the final annotation files through the online software CPJSdraw were analyzed (Li et al., 2023).

### 2.4 Analysis of variation hotspots

The mVISTA program (Frazer et al., 2004) was used in the shuffle-LAGAN mode to precisely align and vividly visualize the boundaries of the inverted repeat (IR) regions in the plastomes of Chinese endemic *Rubus* species. Furthermore, the DnaSP v. 6.10 software (Rozas et al., 2017) was utilized to conduct a meticulous sliding window analysis. Through this analysis, the nucleotide diversity (Pi) of the chloroplast genomes was calculated. The window length was set to 600 base pairs (bp), and the step size was configured to 200 bp. Based on these results, the positions with high Pi values were selectively identified.

### 2.5 Analyses of codon usage bias and selection pressure

To analyze codon usage patterns, we first processed the coding sequences using the RSCU (relative synonymous codon usage) module of CPStools (Huang et al., 2024). Sequences were filtered to remove duplicates, those shorter than 200 bp (to ensure sufficient codon sampling), and transcripts lacking an ATG start codon. Merged sequences were subsequently analyzed for codon usage bias, with results cross-validated using CodonW v1.4.2 (http://codonw.sourceforge.net). and amino acid frequencies were analyzed using CGAS (Abdullah et al., 2025).

To assess the influence of selective pressures on *Rubus* evolution, we performed pairwise Ka/Ks analysis using the Ka/Ks module of CPStools.

Protein-coding genes were extracted using the phy module of CPStools (Huang et al., 2024) and subsequently multiple sequence alignment was performed with MAFFT software (Katoh et al., 2013). Then the aligned protein sequences were reverse-mapped to their corresponding nucleotide sequences and the alignment results were converted into AXT format to calculate the Ka/Ks ratios using KaKs_Calculator 2.0 software.

To further elucidate the characteristics of selective pressure, HyPhy v2.5.7 software (Pond et al., 2016) was used to perform analyses via two approaches: the branch-site unrestricted statistical test for episodic diversification (BUSTED) (Murrell et al., 2015) and the Mixed Effects Model of Evolution (MEME) (Murrell et al., 2012). The former was applied to detect gene-wide signatures of episodic positive selection across different phylogenetic branches, while the latter was used to identify site-specific episodic diversifying selection acting on a subset of lineages. Meanwhile, codon alignment of each individual gene was conducted using MUSCLE v5 (Edgar, 2022) as a prerequisite step for the aforementioned analyses, and the relevant methods were described in previous studies (Abdullah et al., 2025).

### 2.6 Repeat sequences identification

To comprehensively explore the repeat sequence characteristics within the chloroplast genomes of 48 Chinese *Rubus* species, we harnessed the MISA-web online tool (Beier et al., 2017; https://webblast.ipk-gatersleben.de/misa/). The minimum thresholds for SSR detection were set at 10, 5, 4, 3, 3, and 3 for mononucleotide, dinucleotide, trinucleotide, tetranucleotide, pentanucleotide, and hexanucleotide repeats, respectively.

We used the online program REPuter (Kurtz et al., 2001) (https://bibiserv.cebitec.uni-bielefeld.de/reputer/) to identify forward repeat (F), reverse repeat (R), palindrome repeat (P), and complementary repeat (C) of long repeat sequences in these Chloroplast genomes, that met the requirements of a minimum repeat size of 30 bp and accuracy threshold higher than 90% (Hamming Distance = 3).

### 2.7 Phylogenetic analysis

One hundred and four *Rubus* species distributed in China were collected and used to conduct phylogenetic analysis(**Supplementary Table 1**), including 50 species newly assembled and annotated in this study and 54 species from NCBI (**Table 1**), choosing *G*. *aleppicum* and *F*. *palmata* as outgroups. The representative species of each clade are marked with a superscript “T” in the phylogenetic tree. We used the complete chloroplast genomes and protein-coding regions to analyze the phylogenetic relationships of *Rubus* species, respectively. Before constructing the phylogenetic tree, the chloroplast DNA sequences were aligned using MAFFT software (Nakamura et al., 2018) with default parameters. And then, trim the aligned sequences using the trimAl tool (Capella-Gutiérrez et al., 2009) to remove low-quality segments and retain conserved sequences.

**Table 1:** Basic characteristics of the chloroplast genomes of Chinese endemic *Rubus* species.

Species	Length(bp)				GC content (%)						
	Complete	LSC	IR	SSC	Total	CDS	tRNA	rRNA	IR	LSC	SSC
*R. stipulosus*^T^ T. T. Yu & L. T. Lu	156247	85832	25771	18873	37.98	37.98	45.07	55.42	42.80	35.11	31.22
*R. quinquefoliolatus* ^T^ T. T. Yu & L. T. Lu	156395	86062	25763	18807	37.95	37.96	45.13	55.42	42.77	35.06	31.19
*R. menglaensis* ^T^ T. T. Yu & L. T. Lu	156307	85896	25772	18867	37.96	37.96	45.09	55.42	42.79	35.09	31.16
*R. foliaceistipulatus*^T^ T. T. Yu & L. T. Lu	156247	85826	25778	18865	37.91	37.92	45.10	55.42	42.75	35.04	31.09
*R. lucens*^T^ Focke	156031	85699	25778	18776	37.85	37.88	44.92	55.42	42.77	34.88	30.98
*R. luchunensis*^T^ T. T. Yu & L. T. Lu	155988	85550	25772	18894	37.97	37.97	45.05	55.42	42.78	35.13	31.20
*R. metoensis* ^T^ T. T. Yu & L. T. Lu	156450	86127	25766	18791	37.95	37.96	45.13	55.42	42.79	35.01	31.20
*R. neofuscifolius*^T^ Y. F. Deng	156372	85989	25761	18861	37.97	37.98	45.05	55.42	42.78	35.11	31.21
*R. tsangorum*^T^ Hand.-Mazz.	156237	85865	25761	18850	37.97	37.97	45.08	55.42	42.78	35.11	31.21
*R. calycinus*^T^ Wall. ex D. Don	155781	85506	25719	18837	37.94	37.95	45.04	55.39	42.77	35.05	31.17
*R. idaeus* var. *borealisinensis*^T^ T. T. Yu & L. T. Lu	155687	85028	25970	18719	38.02	38.00	45.03	55.42	42.81	35.16	31.30
*R. saxatilis*^T^ L.	156870	86370	25814	18872	37.91	37.94	45.07	55.42	42.74	35.00	31.16
*R. caesius*^T^ L.	156886	86067	25973	18873	37.93	37.98	45.04	55.44	42.82	34.98	31.11
*R. chamaemorus*^T^ L.	156800	85930	26000	18870	38.00	38.00	45.06	55.46	42.83	35.14	31.21
*R. gongshanensis* ^T^	156315	85917	25783	18832	37.97	37.97	45.08	55.42	42.78	35.10	31.23
*R.nyalamensis*^T^ T. T. Yu & L. T. Lu	156340	86015	25759	18807	37.97	37.98	45.11	55.42	42.78	35.05	31.26
R. ellipticus* Sm.	155671	85388	25781	18721	37.95	37.94	44.93	55.39	42.83	35.03	31.12
R. wallichianus* Wight & Arn.	155540	85310	25769	18692	37.97	37.95	44.94	55.39	42.83	35.06	31.14
*R. ellipticus* var. obcordatus* (Franch.) Focke	155659	85392	25769	18729	37.95	37.94	44.91	55.39	42.83	35.02	31.12
R. ichangensis* Hemsl. & Kuntze	156316	85905	25777	18857	37.96	37.96	45.06	55.42	42.78	35.09	31.20
R. lasiotrichos*	156255	85863	25771	18850	37.98	37.98	45.08	55.42	42.79	35.12	31.21
R. crataegifolius* Bunge	155695	85400	25788	18719	37.91	37.92	44.92	55.39	42.81	34.97	31.05
R. flosculosus* Focke	156005	85189	26000	18816	38.01	38.02	45.13	55.41	42.78	35.21	31.26
R. lasiostylus* Focke	155819	85086	26010	18713	38.00	37.98	45.14	55.42	42.79	35.22	31.21
R. subinopertus* T. T. Yu & L. T. Lu	155472	84843	26009	18611	38.03	37.98	45.05	55.39	42.79	35.18	31.37
R. hypopitys* Focke	156262	85836	25778	18870	37.97	37.96	45.09	55.42	42.78	35.11	31.19
R. lineatus* Reinw. ex Blume	156564	86337	25751	18725	37.94	37.97	45.13	55.42	42.75	34.99	31.27
R. paniculatus* Sm.	156267	85856	25781	18849	37.97	37.97	45.05	55.42	42.77	35.10	31.22
R. treutleri* Hook. f.	156263	85858	25771	18863	37.98	37.97	45.09	55.42	42.79	35.11	31.23
R. yunanicus*	156299	85896	25777	18849	37.98	37.97	45.09	55.42	42.79	35.11	31.22
R. kumaonensis* Wall. ex Hook. f.	156244	85852	25771	18850	37.98	37.98	45.07	55.42	42.78	35.11	31.24
R. rufus* Focke	156259	85830	25771	18887	37.97	37.96	45.09	55.42	42.80	35.11	31.17
R. chrysobotrys* Hand.-Mazz.	156272	85884	25761	18866	37.97	37.97	45.05	55.42	42.78	35.12	31.20
R. refractus* H. Lév.	156256	85864	25771	18850	37.97	37.98	45.08	55.42	42.78	35.11	31.22
R. fockeanus* Kurz	156302	85986	25758	18800	37.97	37.98	45.11	55.42	42.79	35.08	31.23
R. faberi* Focke	156226	85849	25771	18835	38.00	37.98	45.07	55.42	42.80	35.12	31.26
R. lobophyllus* Y. K. Shih ex F. P. Metcalf	156259	85862	25774	18849	37.97	37.97	45.06	55.42	42.77	35.12	31.20
R. alceifolius* Poir.	156264	85867	25770	18857	37.97	37.97	45.02	55.42	42.78	35.10	31.21
R. pluribracteatus* L. T. Lu & Boufford	156268	85877	25771	18849	37.97	37.97	45.06	55.42	42.78	35.10	31.23
R. delavayi* Franch.	155603	85270	25752	18829	37.88	37.86	44.82	55.39	42.82	34.95	30.93
R. stans* Focke	155841	85063	25997	18784	38.02	38.04	45.11	55.41	42.79	35.24	31.26
R. niveus* Wall. ex G. Don	155971	85198	25996	18781	38.01	38.02	45.12	55.41	42.80	35.20	31.25
R. parkeri* Hance	156365	85953	25777	18858	37.97	37.97	45.09	55.42	42.79	35.10	31.19
R. coreanus* Miq.	155793	85058	25992	18751	38.04	38.06	45.09	55.41	42.79	35.22	31.36
R. peltatus* Maxim.	155582	85329	25737	18779	37.78	37.81	44.82	55.37	42.81	34.69	30.80
R. rosifolius* Sm.	155655	85449	25748	18710	37.84	37.88	44.89	55.39	42.85	34.77	30.87
R. setchuenensis* Bureau & Franch.	156295	85879	25771	18874	37.98	37.98	45.07	55.42	42.80	35.11	31.22
R. cochinchinensis* Tratt.	156240	85850	25771	18848	37.98	37.98	45.08	55.42	42.78	35.12	31.23
*R. amabilis* Focke	155279	84544	25991	18753	37.31	37.92	45.04	55.37	42.79	35.25	31.40
*R. arcticus* L.	156668	85958	25977	18756	37.17	37.92	45.07	55.42	42.80	35.08	31.20
*R. bambusarum* Focke	156309	85880	25794	18841	37.17	37.92	45.09	55.42	42.80	35.10	31.23
*R. biflorus* Buch.-Ham. ex Sm.	155810	85079	26010	18711	37.27	37.93	45.13	55.42	42.79	35.23	31.23
*R. buergeri* Miq.	156151	85761	25771	18848	37.16	37.63	45.04	55.42	42.78	35.09	31.24
*R. caesius* L.	156886	86067	25973	18873	37.11	37.92	45.03	55.44	42.82	34.98	31.11
*R. calophyllus* C. B. Clarke	156406	86037	25749	18871	37.10	37.92	45.05	55.42	42.75	35.03	31.14
*R. calycacanthus* H. Lév.	156273	85849	25782	18860	37.18	37.91	45.07	55.42	42.78	35.13	31.20
*R. calycinus* Wall. ex D. Don	155781	85506	25719	18837	37.13	37.88	45.03	55.39	42.77	35.05	31.17
*R. chingii* Hu	155563	85322	25749	18743	37.06	37.79	44.85	55.42	42.84	34.94	30.83
*R. columellaris* Tutcher	156123	85761	25779	18804	37.04	37.85	44.92	55.42	42.78	34.91	31.06
*R. crassifolius* T. T. Yu & L. T. Lu	156241	85834	25777	18853	37.17	37.78	45.07	55.43	42.78	35.12	31.18
*R. crataegifolius* Bunge	155714	85402	25781	18750	37.11	37.85	44.91	55.39	42.82	35.00	31.06
*R. ellipticus* Sm.	155655	85390	25769	18727	37.14	37.88	44.90	55.39	42.83	35.03	31.10
*R. ellipticus* var. *obcordatus* Focke	155671	85404	25769	18729	37.14	37.87	44.89	55.39	42.83	35.02	31.12
*R. eucalyptus* Focke	155672	85312	25748	18864	37.06	37.84	44.82	55.42	42.84	34.92	30.94
*R. foliaceistipulatus* T. T. Yu & L. T. Lu	156247	85826	25778	18865	37.11	37.88	45.09	55.42	42.75	35.04	31.09
*R. neofuscifolius* Y. F. Deng	156372	85989	25761	18861	37.17	37.92	45.04	55.42	42.78	35.11	31.21
*R. henryi* Hemsl. & Kuntze	158953	88586	25770	18827	37.09	37.91	44.64	55.42	42.80	35.01	31.23
*R. hirsutus* Thunb.	156380	86934	25369	18708	37.00	37.79	44.88	55.42	42.85	34.87	31.02
*R. hunanensis* Hand.-Mazz.	156217	85806	25790	18831	37.18	37.91	45.06	55.42	42.76	35.13	31.20
*R. idaeus* var. *borealisinensis* T. T. Yu & L. T. Lu	155687	85028	25970	18719	37.25	37.92	45.01	55.42	42.81	35.16	31.30
*R. irenaeus* Focke ex Diels	156232	85814	25771	18876	37.18	37.92	45.07	55.42	42.80	35.11	31.20
*R. irritans* Focke	155286	84613	25988	18697	37.29	37.92	45.05	55.39	42.81	35.21	31.37
*R. jambosoides* Hance	155480	85300	25749	18682	37.06	37.80	44.84	55.42	42.84	34.91	30.94
*R. kawakamii* Hayata	155935	85512	25790	18843	37.17	37.90	45.01	55.45	42.76	35.12	31.18
*R. lambertianus* Ser.	156321	85883	25782	18874	37.17	37.92	45.06	55.42	42.78	35.12	40.52
*R. lambertianus* var. glaber Hemsl.	156263	85883	25782	18816	37.16	37.83	44.77	55.46	38.26	35.10	31.18
*R. leucanthus* Hance	156068	85535	25885	18763	37.03	37.80	44.98	55.42	42.79	34.88	30.98
*R. lucens* Focke	156031	85699	25778	18776	37.02	37.81	44.91	55.42	42.77	34.88	30.98
*R. luchunensis* T. T. Yu & L. T. Lu	155988	85550	25772	18894	37.18	37.97	44.05	55.42	42.78	35.13	31.20
*R. menglaensis* T. T. Yu & L. T. Lu	156307	85896	25772	18867	37.16	37.90	45.08	55.42	42.79	35.09	31.16
*R. metoensis* T. T. Yu & L. T. Lu	156450	86127	25766	18791	37.11	37.92	45.12	55.42	42.79	35.01	31.20
*R. pacificus* Hance	156255	85864	25771	18849	37.17	37.91	45.08	55.42	42.78	35.11	31.22
*R. pectinaris* Focke	156506	86214	25753	18786	37.12	37.81	45.06	55.43	42.77	35.01	31.25
*R. pentagonus* Wall. ex Focke	156287	85958	25763	18803	37.16	37.94	44.80	55.46	42.77	35.09	31.20
*R. pileatus* Focke	155464	84847	25997	18623	37.27	37.90	45.03	55.39	42.79	35.18	31.41
*R. poliophyllus* Kuntze	156298	85894	25777	18850	37.16	37.74	45.06	55.42	42.79	35.09	31.20
*R. reflexus* var. *lanceolobus* F. P. Metcalf	156238	85847	25771	18849	37.18	37.92	45.07	55.42	42.78	35.12	31.24
*R. refractus* H. Lév.	156256	85864	25771	18850	37.17	37.91	45.07	55.42	42.78	35.11	31.22
*R. saxatilis* L.	156870	86370	25814	18872	37.09	37.91	45.06	55.42	42.74	35.00	31.16
*R. setchuenensis* Bureau & Franch.	156295	85879	25771	18874	37.17	37.92	45.06	55.42	42.80	35.11	31.22
*R. stipulosus* T. T. Yu & L. T. Lu	156247	85832	25771	18873	37.18	37.92	45.06	55.42	42.80	35.11	31.22
*R. subinopertus* T. T. Yu & L. T. Lu	155472	84843	26009	18611	37.27	37.88	45.03	55.39	42.79	35.18	31.37
*R. sumatranus* Miq.	155935	85662	25772	18729	37.03	37.83	44.91	55.39	42.83	34.85	31.04
*R. swinhoei* Hance	156335	85897	25790	18858	37.16	37.91	45.08	55.42	42.76	35.10	31.16
*R. taitoensis* Hayata	155948	85175	25992	18789	37.27	37.97	45.09	55.41	42.79	35.22	31.30
*R. taiwanicola* Koidz. & Ohwi	155616	85408	25742	18724	36.98	37.78	44.90	55.39	42.86	34.78	30.83
*R. tephrodes* Hance	156217	85806	25790	18831	37.18	37.91	45.06	55.42	42.76	35.13	31.20
*R. tsangii* Merr.	155995	85758	25764	18709	37.01	37.65	44.81	55.45	42.86	34.80	31.04
*R. tsangiorum* Hand.-Mazz.	156237	85865	25761	18850	37.17	37.91	45.07	55.42	42.78	35.11	31.21
*R. wallichianus* Wight & Arn.	155562	85333	25769	18691	37.15	37.85	44.93	55.39	42.83	35.04	31.16
*R. xanthocarpus* Bureau & Franch.	156052	85653	25762	18875	37.06	37.72	44.81	55.43	42.83	34.94	30.98
*R. xanthoneurus* Focke ex Diels	156300	85900	25773	18854	37.17	37.77	45.07	55.43	42.78	35.12	31.20

The IQ-TREE 2 software (Quang et al., 2020) was employed to select the optimal base substitution model, and the TVM+F+R4 model (complete chloroplast genomes) and TVM+F+R3 model (protein-coding regions) were selected. A phylogenetic tree was then constructed with a bootstrap value set at 1000. Finally, we used the TVBOT tool (Xie et al., 2023) (https://www.chiplot.online/) for tree visualization and edition.

In this study, the length variation of the inverted repeat (IR) region and the phylogenetic relationships among *Rubus* species were studied. We calculated the IR length for each species and constructed a phylogenetic tree-like diagram with reference to an R script (Abdullah et al., 2025). IR lengths were mapped onto the phylogenetic tree using a color gradient to visualize patterns of length variation across different clades.

## 3 Results

### 3.1 Basic characteristics of *Rubus* chloroplast genomes

**Figure 1:**
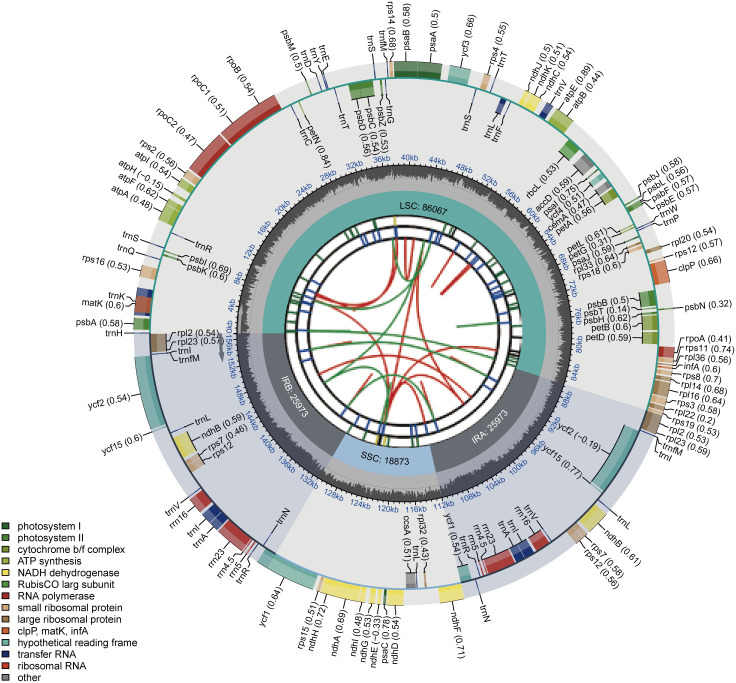
Chloroplast genome map of *Rubus.* The map contains six tracks in default. From the center outward, the first track shows the dispersed repeats. The dispersed repeats consist of direct (D) and Palindromic (P) repeats, connected with red and green arcs. The second track shows the long tandem repeats as short blue bars. The third track shows the short tandem repeats or microsatellite sequences as short bars with different colors. The colors, the type of repeat they represent, and the description of the repeat types are as follows. Black: c (complex repeat); Green: p1 (repeat unit size = 1); Yellow: p2 (repeat unit size = 2); Purple: p3 (repeat unit size = 3); Blue: p4 (repeat unit size = 4); Orange: p5 (repeat unit size = 5); Red: p6 (repeat unit size = 6). The small single-copy (SSC), inverted repeat (IRa and IRb), and large single-copy (LSC) regions are shown on the fourth track. The GC content along the genome is plotted on the fifth track. The genes are shown on the sixth track.The values of codon bias are shown in parentheses after the gene names. Genes are color - coded according to their functional classification. The transcription directions of inner and outer genes are clockwise and counterclockwise respectively. The functional classification of genes is shown in the lower left corner.

We sequenced forty-eight *Rubus* species and assembled their complete chloroplast genomes. It was found that the chloroplast genomes of all the species exhibited a typical circular quadripartite structure: Two inverted repeat regions (IRa and IRb) separate the large single-copy region (LSC) from the small single-copy region (SSC), and no substantial rearrangement was detected (**Figure 1**). The complete chloroplast genomes of these Chinese *Rubus* species ranged from 155,472 bp (*R*. *subinopertus*) to 156,886 bp (*R*. *caesius*), with 37.78% - 38.04% GC content. The length of the LSC region is 84,843 - 86,370 bp, the SSC region is 18,611 - 18,894 bp, and the IRs region is 25719–26010 bp (**Table 1**).

These chloroplast genomes were conserved not only in their structure but also in the number of different coding genes. Each species contained 88 protein-coding genes (*PCG*s), 8 rRNA genes and 35 tRNA genes, and the coding genes were functionally classified into photosynthesis-related genes (45), self-replication-related genes (72), and other genes (14). Genes with two exons included *ndhA, ndhB, petB, petD, rpl16, rpl2, rpoC1, rps16, trnA-UGC, trnI-GAU, trnK-UUU, trnL-UAA*, and *trnV-UAC*, while genes with three exons comprised *clpP, rps12*, and *ycf3* (**Supplementary Table 2**). In this study, 18 intron-containing genes were identified from the set of coding genes, among which, 12 genes were protein-coding genes and 6 genes were tRNA genes. The distribution positions of these intron-containing genes were highly conserved across all the investigated *Rubus* species. Among the protein-coding sequences, the intron of the *rps12* gene had the shortest length and the most conservation, ranging from 510 to 545 bp. In contrast, the intron of the *ndhA* gene was the longest among all intron-containing genes, with a length range of 1189 to 1314 bp. Other introns that exhibited significant length variation included those of the *petB* and *petD* genes (**Supplementary Table 2**).

### 3.2 Comparisons and analyses of the IR boundaries

**Figure 2:**
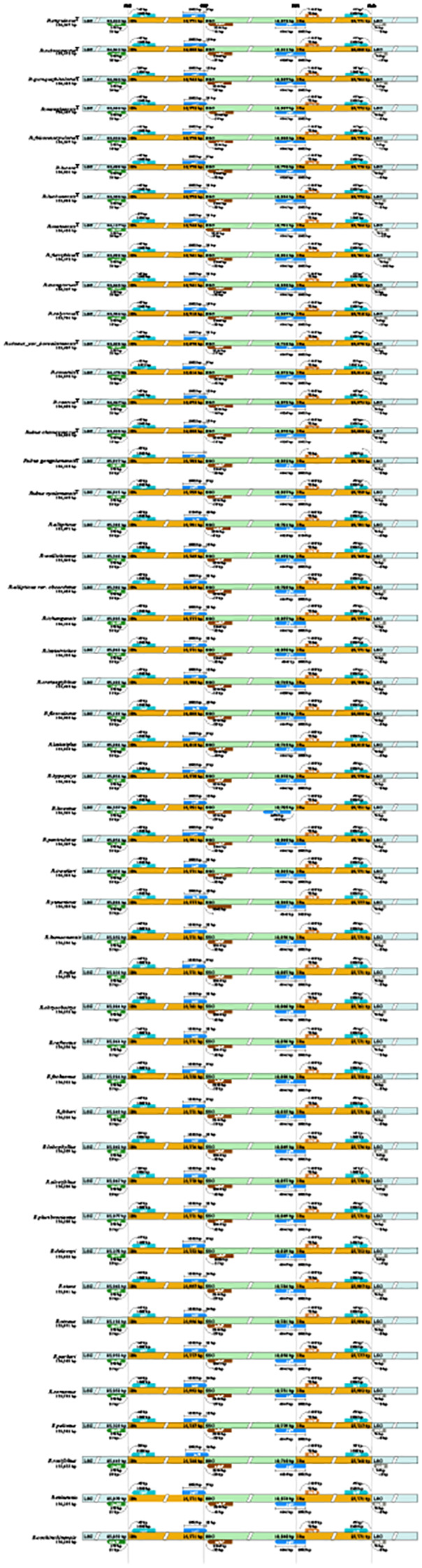
Boundary analysis of Chinese *Rubus* species.

We analyzed the expansion and contraction of the IRs region in the chloroplast genomes of 48 Chinese *Rubus* species and found that the boundaries between the IR regions and single-copy regions exhibit a high degree of conservation (**Figure 2**). The length of the IRs region ranges from 25,719 to 26,010 bp. The JLB (LSC/IRb)boundary is situated between the *rps19* and *rpl2* genes, and *rps19* is 11 to 24 bp away from the JLB boundary. As for the JLA boundary, it lies between the *trnH* and *rpl2* genes, with *trnH* being 0 to 12 bp distant from the JLA boundary. Notably, in *R*. *neofuscifolius*, the distance between *trnH* and the JLA boundary measures is 105 bp.

Since IRa and IRb are inverted repeat regions, the length between *rpl2* and the JLB/JLA(IRa/LSC)boundary ranges from 37 to 52 bp. Additionally, the JSA (SSC/IRa) and JSB (SSC/IRb) boundaries of all Chinese *Rubus* species are located within the *ycf1* gene examined *R. lineatus* Reinw. ex Blume. The length of the *ycf1* gene remains consistent with the IRa and IRb regions. However, the length of SSC region ranges from 4638 to 4668 bp, which may account for the slight discrepancies in the chloroplast genome sizes among the *Rubus* species.

### 3.3 Analysis of variation hotspots

**Figure 3:**
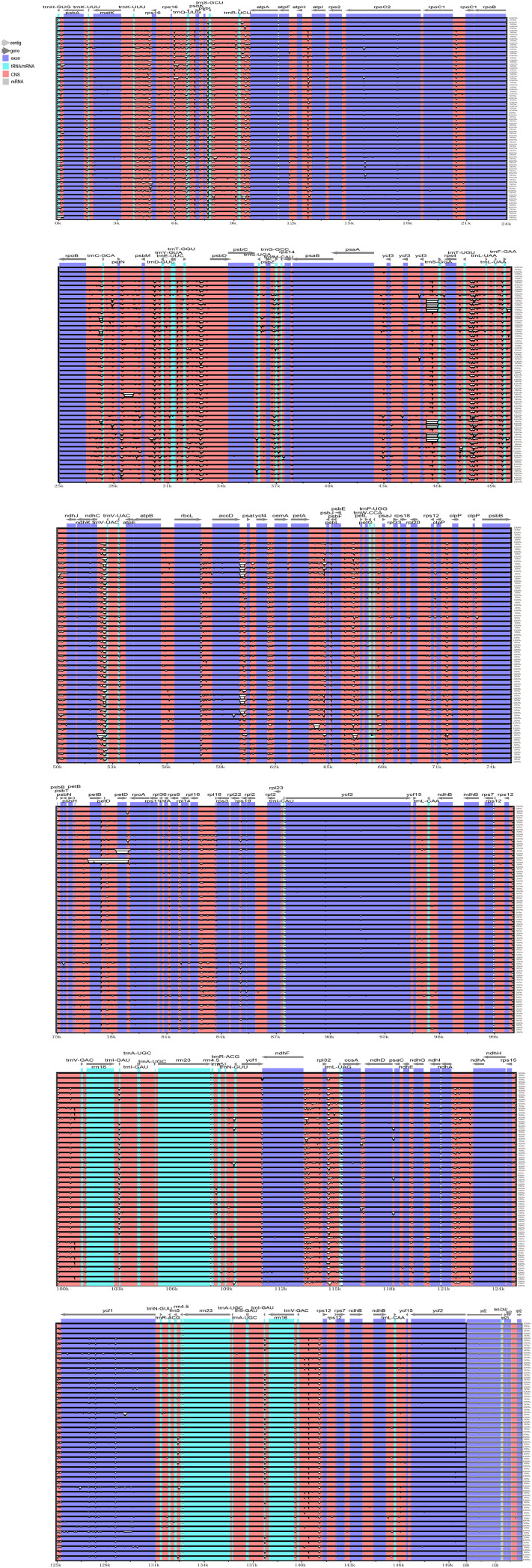
Comparative analysis of chloroplast genome sequences of Chinese *Rubus* species. *R*. caesius was used as the reference genome. Genes are represented by gray arrows at the top of the sequences, and different genes are indicated by various colors. The vertical axis represents the consistency ranging from 50% to 100%. The representative Chinese *Rubus* species from top to bottom in the figure 3. are *R*. *ellipticus*, *R*. *wallichianus*, *R*. *ellipticus* var. *obcordatus*, *R*. *ichangensis*, *R*. *stipulosus*, *R*. *lasiotrichos*, *R*. *crataegifolius*, *R*. *flosculosus*, *R*. *lasiostylus*, *R*. *subinopertus*, *R*. *hypopitys*, *R*. *quinquefoliolatus*, *R*. *menglaensis*, *R*. *lineatus*, *R*. *foliaceistipulatus*, *R*. *paniculatus*, *R*. *treutleri*, *R*. *yunanicus*, *R*. *lucens*, *R*. *kumaonensis*, *R*. *luchunensis*, *R. metoensis*, *R*. *rufus*, *R*. *fuscifolius*, *R*. *chrysobotrys*, *R*. *refractus*, *R*. *tsangiorum*, *R*. *fockeanus*, *R*. *calycinus*, *R*. *faberi*, *R*. *lobophyllus*, *R*. *alceifolius*, *R*. *pluribracteatus*, *R*. *delavayi*, *R*. *stans*, *R*. *niveus*, *R*. *parkeri*, *R*. *coreanus*, *R*. *idaeus* var. *borealisinensis*, *R*. *saxatilis*, *R*. *peltatus*, *R*. *caesius*, *R*. *rosifolius*, *R*. *setchuenensis*, *R*. *cochinchinensis*, *R*. *chamaemorus*, *R*. *gongshanensis*, *R*. *nyalamensis*.

**Figure 4:**
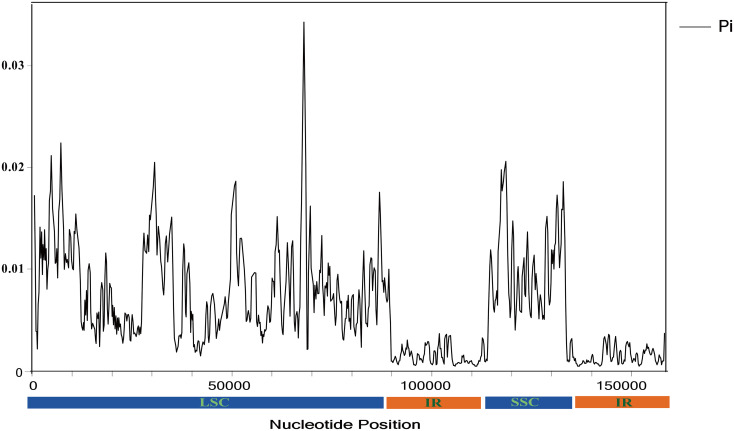
Comparison of nucleotide diversity (Pi) values among Chinese *Rubus* species (window length: 600 bp, step size: 200 bp). X-axis, position of the midpoint of each window; Y-axis, nucleotide diversity (pi) of each window.

Using the sequence of *R. caesius* as a reference, the chloroplast genome sequences of Chinese *Rubus* species were compared by using the mVISTA program (Frazer et al., 2004) (http://genome.lbl.gov/vista/mvista/about.shtml) (**Figure 3**) The results showed that LSC region and the small single-copy (SSC) region exhibited greater variation than the inverted repeat (IR) region.(**Figures 3**,**4**).

**Figure 5:**
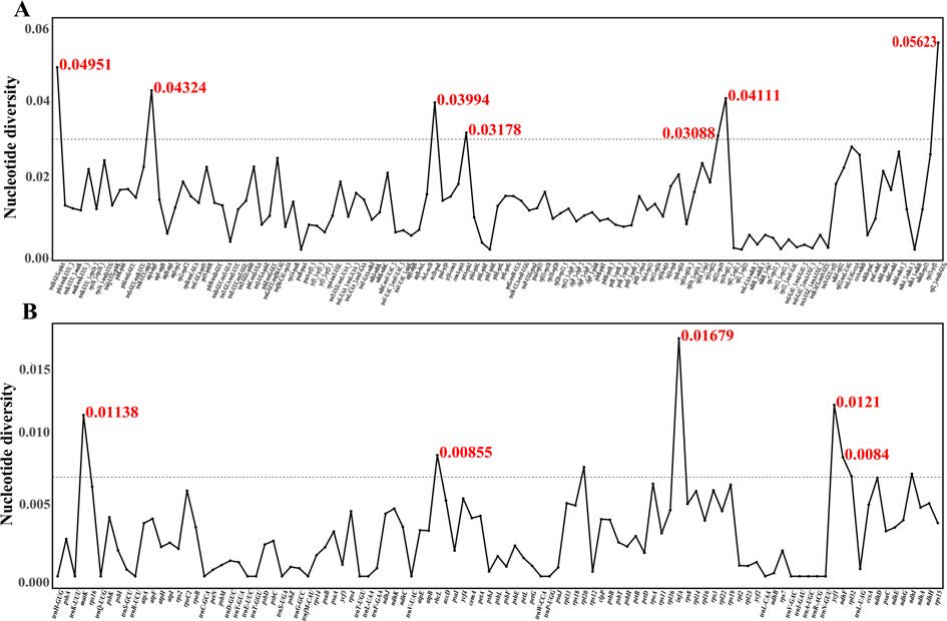
Nucleotide diversity (Pi) values of different regions in the plastomes of Chinese *Rubus* species. Regions with higher Pi values are above the dotted line. **(A)** shows the Pi values of non-coding regions, and **(B)** shows the Pi values of protein-coding regions.

Based on the sliding window analysis using DnaSP V6 software, the Pi value of Chinese *Rubus* species was calculated. The variation in non-coding regions was greater than in coding regions (**Figures 5A, B** and **Supplementary Table 3**). In the coding regions, when the Pi value was > 0.007, and five highly variable regions were found: *rbcL* (0.00855), *matK* (0.01138), *ndhF* (0.00840), *infA* (0.01679) and *ycf1* (0.0121). When the Pi value > 0.03, in the non-coding regions, there were highly variable regions such as *trnH-GUG-psbA*(0.04951), *atpA*-*atpF* (0.04324), *accD-psaI* (0.03994), *petA*-*psbJ* (0.03178), *rpl22*-*rps19* (0.03088), *rps19*-*rpl2*_2(0.04111), *rpl2_2*-*trnH*-*GUG*(0.05623).

### 3.4 Repeat sequences analyses

**Figure 6:**
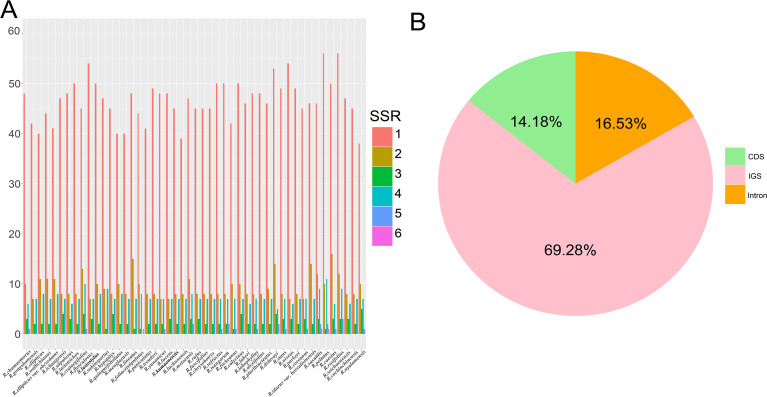
SSRs analyses of complete chloroplast genomes of Chinese *Rubus* species. **(A)** Numbers of SSRs detected in the complete chloroplast genomes of *Rubus* within each species; **(B)** The ratios of SSRs in introns, CDS (coding sequences), and IGS (intergenic regions).

The high polymorphism of SSRs at the species level makes them one of the most commonly used molecular markers in phylogenetic and population genetics studies. A total of 56 (*R*. *luchunensis*) to 76 (*R. rosifolius*) SSRs were detected in the chloroplast genomes of 48 *Rubus* species. Among these SSRs, the majority were mononucleotide repeats (38-56), followed by dinucleotide repeats (7-16), trinucleotide repeats (1-5), tetranucleotide repeats (6-12), pentanucleotide repeats (0-3), and hexanucleotide repeats (0-1) (**Figure 6A**). Mononucleotide repeats may play a more important role in genetic variation than other types of SSRs. SSRs are mainly composed of mononucleotides (A)n and (T)n, which indicates that the base composition of SSRs has a bias towards the use of A/T bases (**Supplementary Table 4**). In addition, the analysis of the locations of SSRs indicates that the SSRs in the chloroplast genomes of Chinese *Rubus* species are mainly distributed in the IGS (intergenic regions) (**Figure 6B**).

**Figure 7:**
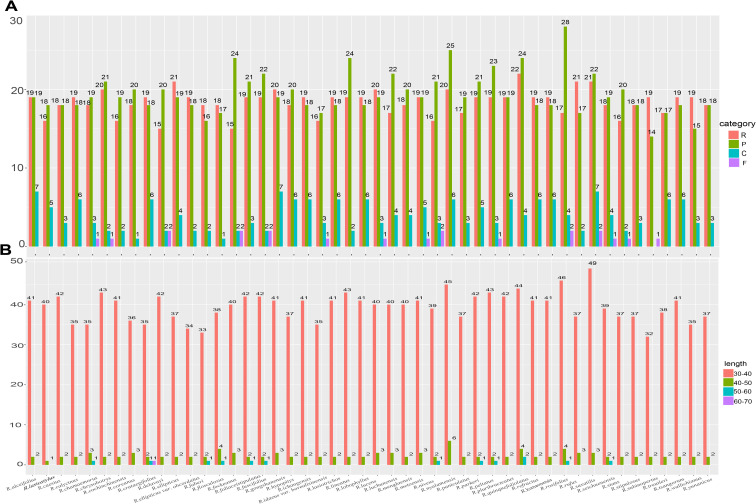
Long repeat sequences analyses of complete chloroplast genomes of Chinese *Rubus* species. **(A)** Numbers of long repeat sequences detected in the complete chloroplast genomes of *Rubus* within each species; **(B)** Numbers of long repeat sequences by length.

Long repeat sequences ≥ 30 bp may promote the rearrangement of the chloroplast genome and enhance the function of species genetic diversity. In the analyses of chloroplast genomes, a total of 2027 long repeat sequences were predicted. The types number of long repeat sequences varies among different Chinese *Rubus* species, ranging from 34 (*R*. *subinopertus*) to 51 (*R*. *nyalamensis* and *R*. *rosifolius* Sm.) which include 15–21 palindromic repeats, 14–28 forward repeats, 0–7 reverse repeats, and 0–2 complementary repeats. Among these repeat sequences, palindromic repeats, forward repeats, and reverse repeats are present in all Chinese *Rubus* species. However, complementary repeats were only detected in *R*. *caesius* (1), *R*. *idaeus* var. *borealisinensis* (1), *R*. *lucens* (1), *R*. *metoensis* (1), *R. lasiostylus* (1), *R. subinopertus* (1), *R. stans* (1), *R. peltatus* (1), *R. setchuenensis* (1) and *R. flosculosus* (2), *R. niveus* (2), *R. coreanus* (2), *R. rosifolius* (2), *R*. *foliaceistipulatus* (2)*, R*. *saxatilis* L. (2) (**Figure 7A**). Moreover, most sequences are distributed between 30 and 40 bp, followed by those between 40 and 50 bp, 50–60 bp (except for *R*. *lucens* and *R*. *saxatilis*). Only *R*. *crataegifolius* contains a long repeat sequence (62 bp) between 60 and 70 bp.(**Figure 7B** and **Supplementary Table 5**).

### 3.5 Analysis of codon usage

**Figure 8:**
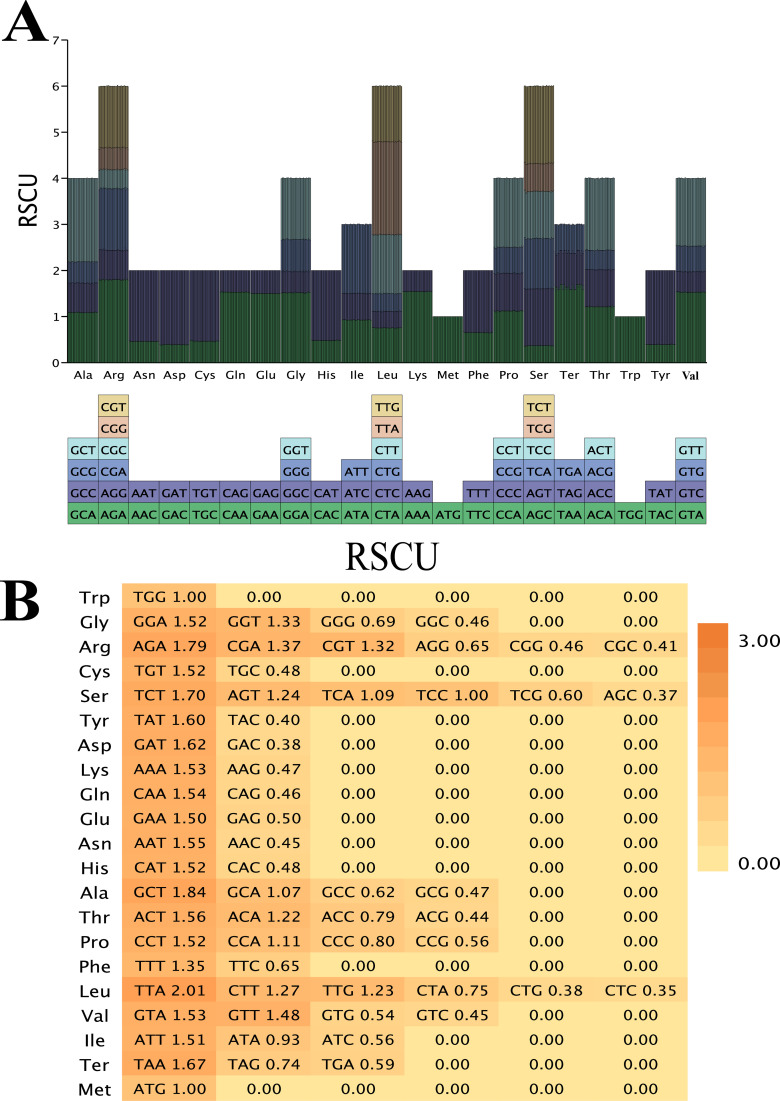
A codon usage bias analysis of complete chloroplast genomes of Chinese *Rubus* species. **(A)** Statistics from RSCU values of amino acids. The histogram corresponds to the same color as the codon. **(B)** Heat - map visualization of relative synonymous codon usage (RSCU) values of amino acids, where the shade of color represents the magnitude of RSCU values.

By analyzing the codon usage bias in the chloroplast genomes of 48 Chinese *Rubus* species, we found that, except for *R. lineatus* with a relatively low codon count (21,611), the codon counts of all other species were concentrated in the range of 22,011–22,038. AUU, which encodes isoleucine, was the most frequently used codon, with its number ranging from 934–960 (**Supplementary Table 6**, **Figure 8A**). The RSCU value of UUA is the highest (2.00 - 2.05) (**Figure 8B**). The amino acid frequencies of *Rubus* exhibited a high degree of similarity: among all amino acids, leucine had the highest coding frequency, followed by isoleucine, while cysteine had the lowest (**Supplementary Table 6**). Among the 33 codons with an RSCU value ≥ 1, those ending with A, U, G, and C account for 39.39%, 48.48%, 9.09%, and 3.03%, respectively. There are 29 codons ending with A or U, accounting for 87.88%. (**Supplementary Table 6**). We can conclude that the preferred codons in the chloroplast genomes of *Rubus* species end with A or U. It is worth noting that the RSCU values of methionine and tryptophan are 1(**Figure 8A**), which indicates that there is no codon bias between them.

### 3.6 Selection analyses

**Figure 9:**
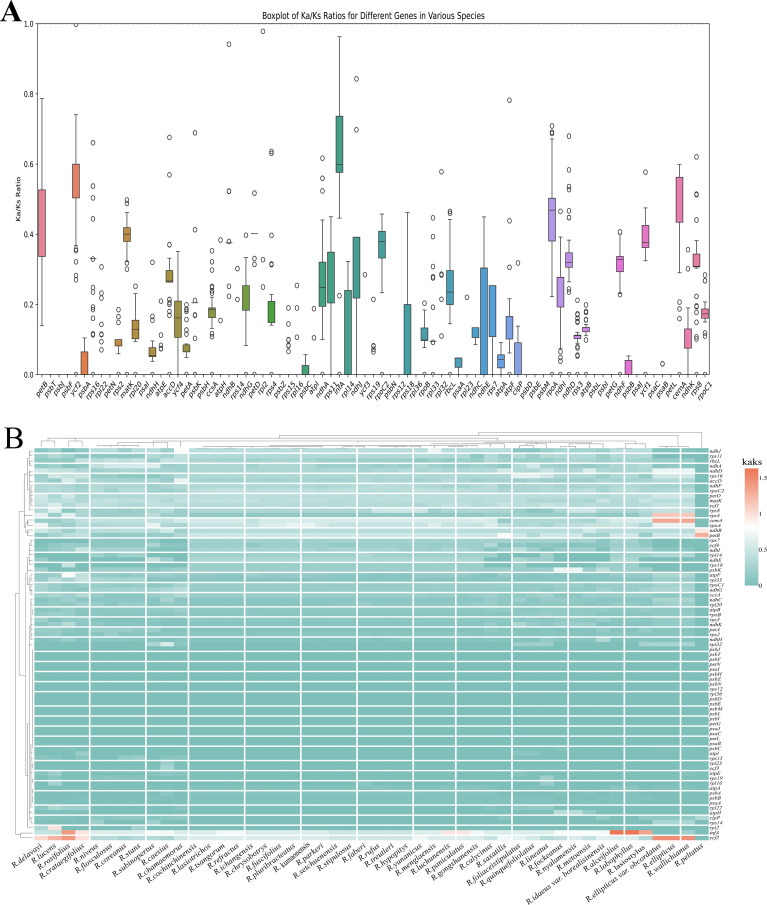
Comparison of the Ka/Ks ratio values of PCGs in complete chloroplast genomes of Chinese *Rubus*, with *R. peltatus* as the reference sequence. **(A)**. visualizes the central tendency and degree of dispersion of different PCGs using box plots. **(B)** visualizes different PCGs of different species using heatmaps.

The chloroplast genome of *R. peltatus* was chosen as the reference sequence. The Ka/Ks variations of the chloroplast (cp) genomes of Chinese *Rubus* species were systematically analyzed (**Figures 9A, B**), and the Ka/Ks ratios of 79 protein-coding genes (PCGs) were calculated. The results showed that the Ka/Ks ratios of these PCGs varied among the *Rubus* species, ranging from 0 to 1.63207. Notably, except for *petA, ycf2, rps4, infA*, and *cemA*, which had Ka/Ks ratios ≥1 in some species (**Supplementary Table 7**), all other *PCG*s had Ka/Ks ratios <1, and some even had no detectable Ka/Ks ratios. This suggests that the former five genes may be undergoing positive selection, while most other genes exhibit high evolutionary stability. To further verify the selection pressure acting on these genes, additional analyses were performed using two modules in HyPhy v2.5.7: BUSTED and MEME. BUSTED, analyses of 48 *Rubus* chloroplast *PCG*s revealed no significant gene-wide episodic positive selection signals (**Supplementary Table 7**). Meanwhile, no valid episodic diversifying selection information was output in the JSON file of MEME analysis. These results indicate that despite the Ka/Ks ratios of a few genes suggesting potential positive selection, the majority of the studied chloroplast *PCG*s are generally under strong purifying selection, thereby reflecting the evolutionary conservation of the *Rubus* chloroplast genome.

### 3.7 Phylogenetic analysis

**Figure 10:**
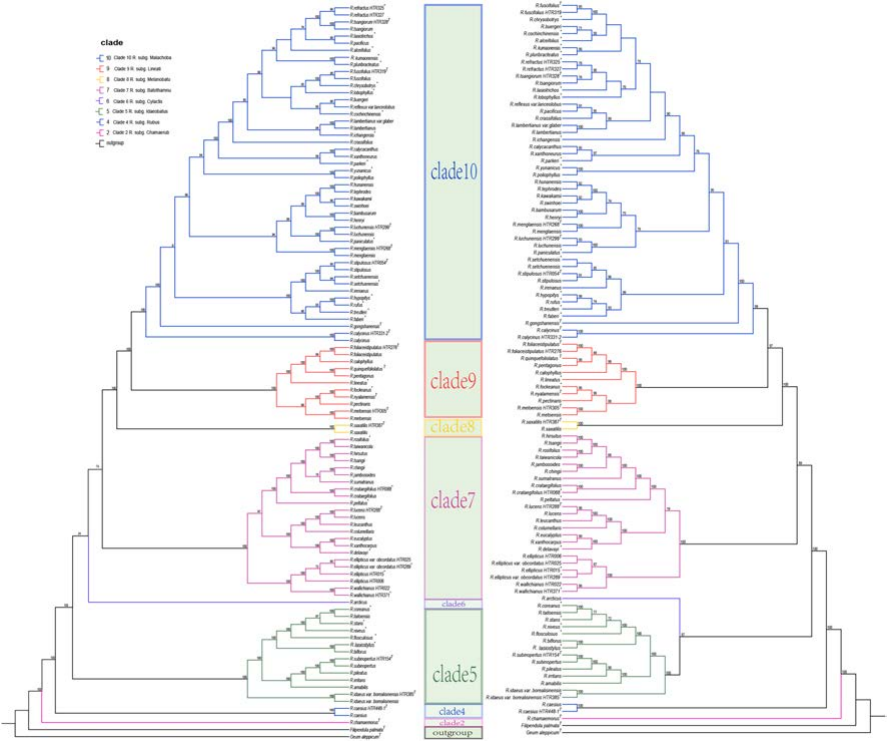
Maximum likelihood phylogenetic tree based on complete chloroplast genomes (right) and maximum likelihood phylogenetic tree based on coding sequences (left).

Phylogenetic analyses based on the complete chloroplast genomes and coding genes of Chinese *Rubus* species identified eight major clades for all *Rubus* samples, with *Filipendula palmata* and *Geum aleppicum* used as outgroups and the clade of *Filipendula* + *Geum* are sister to *Rubus* (**Figure 10**). Among Chinese *Rubus* species, clade 2 and clade 4 are the first two branching clade, which include one species respectively, *Rubus chamaemorus* and *R*. *caesius*. Species of former *R*. subg. *Idaeobatus* Focke with representatives were separated into five clades (clades 5, 6, 7, 8 and 9), and most of these representative species occur in clades 5, 7, and 8, though they are not closely related. Clade 10 contains most species of former *R*. subg. *Malachobatus* (Focke) Fritsch. Species of clade 9 belong to former *R.* subg. *Dalibarda* (L.) Focke, *R*. subg. *Malachobatus* (Focke) Fritsch, *R*. sect. *Cylactis* (Raf.) Focke, *R*. sect. *Idaeobatus* subsect. *Alpestres* (Focke) T. T. Yu & L. T. Lu, *R*. sect. *Malachobatus* subsect. *Foliaceistipulati* T.T.Yu & L.T.Lu, and *R*. sect. *Malachobatus* subsect. *Metoenses* T.T.Yu & L.T.Lu. One species (*R*. arcticus) forms clade 6. These eight distinct clades represent eight subgenera, which correspond to the new classification proposed by Huang et al. (2023), were named clade 2 (*R*. subg. *Chamaerubus*), clade 4 (*R*. subg. *Rubus*), clade 5 (*R*. subg. *Idaeobatus*), clade 6 (*R*. subg. *Cylactis*), clade 7 (*R*. subg. *Batothamnus*), clade 8 (*R*. subg. *Melanobatus*), clade 9 (*R*. subg. *Lineati*), and clade 10 (*R*. subg. *Malachobatus*). In which, The subgenera represented by clade 5, clade 7 and clade 10 may be the three largest subgenera. This research achievement provides a solid and reliable basis for subgeneric classification of *Rubus*, and is helpful for a deeper understanding of phylogenetic and evolutionary relationships among *Rubus* species.

After mapping the lengths of the inverted repeat (IR) regions onto the phylogenetic tree using a color gradient, no significant association was observed between IR length variation and the phylogenetic relationships of *Rubus* species in this study. The IR lengths of *Rubus* species did not exhibit a consistent clustering pattern corresponding to the major clades in the phylogenetic tree. Instead, the variation in IR lengths appeared to be randomly distributed across different clades, and there was no obvious phylogenetic signals (**Supplementary Figure 2**).

## 4 Discussion

Through the assembly, annotation, and a series of comparative analyses of the chloroplast genomes, it was found that chloroplast genomes of *Rubus* exhibit a typical circular quadripartite structure, encompassing LSC, SSC, IRa and IRb. A total of 131 genes were identified in these chloroplast genomes. The size of the complete chloroplast (Cp) genomes ranged from 155472 to 156886 bp. This is consistent with the chloroplast genome size observed in other studies of *Rubus* species (Yu et al., 2022). These chloroplast genomes not only show a high degree of conservation in structure but also in the number of different coding genes. The expansion/contraction of the inverted repeat regions and gene deletions are the main reasons of the chloroplast genome sizes variation among different *Rubus* species (Cheon et al., 2017). The boundary variations are related to the evolution of taxa, so the highly variable genes at the boundaries may serve as evolutionary markers (Qin et al., 2025). There are some minor differences among these Chinese *Rubus* species, and there are no obvious contraction or expansion, which fully indicates that the chloroplast genomes of *Rubus* species are highly stable. This is similar to the situation observed in the chloroplast genomes of other *Rubus* species (Lu et al., 2024).

The results of the sliding window analysis show that the nucleotide Pi in the non - coding regions is higher than that in the coding regions. Especially, the variation degrees in LSC region and the small single - copy (SSC) region are significantly greater than those in the IR region. The studies in the genus *Salix* also support this conclusion (Yuan et al., 2025), such as the *rps16* - *trnQ* - *UUG* - *psbK*, *petN* - *psbM*, *trnF* - *GAA* - *ndhJ*, and *ndhD* - *psaC* regions. In the coding regions, when the Pi value > 0.007, five highly variable regions, *infA*, *matK*, *ndhF*, *rbcL* and *ycf1*, were found. When the Pi value > 0.03, there are seven highly variable regions in the non - coding regions, namely *trnH* - *GUG* - *psbA*, *atpA* - *atpF*, *accD* - *psaI*, *petA* - *psbJ*, *rpl22* - *rps19*, *rps19-rpl2*_2 and *rpl2_2* - *trnH* - *GUG*. These highly variable regions and variable regions are expected to serve as effective molecular markers in phylogenetic research and DNA barcoding research.

In the analysis of simple sequence repeats, the proportion of mononucleotide repeats is the highest. This conclusion is consistent with that of Yu et al. (2022), which suggests that they may play a more crucial role in genetic variation than other types of simple sequence repeats. Simple sequence repeats are mainly composed of mononucleotides (A)n and (T)n, which indicates that the base composition of simple sequence repeats has an obvious bias towards the use of A/T bases. This is consistent with the characteristics of plants in some other family, such as Legumes (Qin et al., 2025). In addition, simple sequence repeats are mainly distributed in intron regions. Although the number of long - repeat sequences varies among different species, their distribution patterns are relatively consistent. In the analysis of codon usage, the RSCU value of the codon UUA encoding leucine is the highest, which is consistent with the conclusions of previous studies (Jiang et al., 2025). Among the 33 codons with an RSCU value ≥ 1, codons ending with A or U account for up to 87.88%, Meanwhile, the RSCU values of methionine and tryptophan are both 1, which indicate that there is no codon bias in the encoding of these two amino acids.

It was found that the Ka/Ks values of *petA, ycf2, rps4, infA*, and *cemA* were > 1 in some species. This suggested that these genes may have undergone positive selection. This not only provides clues for exploring functionally important chloroplast genes, but also highlights their value in plant evolutionary studies. For example, *ycf2* exhibits divergence among different plant groups. Although its exact function remains unclear, it is presumed that it plays a role in photosynthesis, p.

lant growth and development and phylogenetic analyses. In a previous study of eight *Rubus* species from Taiwan, 29 genes with dN/dS > 1 were detected (Yang et al., 2021), which implied that genes involved in various plastid activities may be under positive selection. Although the Ka/Ks ratios of the five genes are greater than 1, BUSTED and MEME did not detected significant positive selection signals. This may reflect false positives caused by individual divergent sites or weak and transient selection signals, which could not be identified by strict statistical methods. In a word, chloroplast genomes of *Rubus* are mainly subjected to strong purifying selection, consistent with their high conservation. Our results further indicate that Ka/Ks ratios can be influenced by reference sequences and analytical methods, and should not be used alone. Comprehensive verification using BUSTED and MEME is essential for reliable evolutionary inferences.

Phylogenetic analysis indicates that these Chinese *Rubus* species were classified into 8 clades, which is consistent with the studies of Phylogeny of *Rubus* (Rosaceae): Integrating molecular and morphological evidence into an infrageneric revision (Huang et al., 2023). However, the branch support rates on the complete chloroplast phylogenetic tree were higher than those on the *PCG*s phylogenetic tree. This result is consistent with the research conclusions on *Aconitum* plants in previous articles (Xia et al., 2022). Phylogenetic trees based on chloroplast genomes and CDS indicates that: there are some sister groups are commonly supported, for example, *Rubus idaeus* var. *borealisinensis* and *R. subinopertus* in clade 5; *Rubus lucens* and *R. peltatus* in clade 7; and *Rubus neofuscifolius* and *R. tsangorum* in clade 10. In other words, Chinese *Rubus* species could be divided into eight subgenera (Huang et al., 2023), which is basically in line with the division of eight groups made by Yu and Lu (1985). However, the scopes of these subgenera have changed compared to the previous divisions, such as *R*. *saxatilis* and *R*. pungens are moved from *R*. sect. *Cylactis* Focke and *R*. sect. *Idaeobatus* Focke respectively, to *R*. subg. *Melanobatus* (Greene) House. Some taxonomic groups with palmately compound leaves, *R*. *metoensis*, *R*. *nyalamensis*, *R. quiquefoliolatus* and *R*. *foliaceistipulatus*, are classified into a new subgenus, *R*. subg. *Lineati* (Focke) T.R.Huang & X.Y.Zhu (Huang et al., 2023).

## 5 Conclusion

In this study, through the assembly, annotation, and in-depth analysis of the chloroplast genomes of Chinese *Rubus* species, the results showed that the chloroplast genomes of *Rubus* exhibited a high degree of conservation, with only subtle differences in aspects such as boundary lengths and the value of Ka/Ks. The chloroplast genomes of Chinese *Rubus* species belong to 8 different subgenera. Meanwhile, this study has successfully identified 12 highly variable regions. These results can provide strong support for the subsequent phylogenetic studies of *Rubus* species, and detailed data evidence for the related research on searching for DNA barcodes and screening effective molecular markers.

However, it should be noted that due to extensive interspecific hybridization, polyploidization, and apomixis, the morphological variations of *Rubus* species are extremely complex, and it is recognized as a notoriously challenging group in plant taxonomy. In recent years, chloroplast genome data have provided molecular evidence for resolving the phylogenetic relationships within *Rubus* and have proposed a subgeneric classification system based on both morphological and molecular evidence. In order to further address the issues related to the origin and differentiation of the *Rubus* species, and to construct a more detailed sectional classification system under the subgenus, various types of data such as morphological, nuclear genome, cytological, and palynological data are required.

## Data Availability

The datasets presented in this study can be found in online repositories. The names of the repository/repositories and accession number(s) can be found below: https://www.ncbi.nlm.nih.gov/genbank/, OP747328.1 https://www.ncbi.nlm.nih.gov/genbank/, OP747329.1 https://www.ncbi.nlm.nih.gov/genbank/, OP747334.1 https://www.ncbi.nlm.nih.gov/genbank/, OP747336.1 https://www.ncbi.nlm.nih.gov/genbank/, OP747409.1 https://www.ncbi.nlm.nih.gov/genbank/, OP747393.1 https://www.ncbi.nlm.nih.gov/genbank/, OP747405.1 https://www.ncbi.nlm.nih.gov/genbank/, OP747394.1 https://www.ncbi.nlm.nih.gov/genbank/, OP747395.1 https://www.ncbi.nlm.nih.gov/genbank/, OP747399.1 https://www.ncbi.nlm.nih.gov/genbank/, OP747401.1 https://www.ncbi.nlm.nih.gov/genbank/, OP747403.1 https://www.ncbi.nlm.nih.gov/genbank/, OP747404.1 https://www.ncbi.nlm.nih.gov/genbank/, OP747365.1 https://www.ncbi.nlm.nih.gov/genbank/, OP747370.1 https://www.ncbi.nlm.nih.gov/genbank/, OP747373.1 https://www.ncbi.nlm.nih.gov/genbank/, OP747374.1 https://www.ncbi.nlm.nih.gov/genbank/, OP747377.1.
