# Supplementary figures and images for: Comparative genomics on chloroplasts of Chinese Rubus: genetic structure and phylogenetic relationships with other species of Rosaceae
Source: Front Plant Sci. 2026 Mar 20;17:1765373. doi: 10.3389/fpls.2026.1765373 (PMC13047156; doi:10.3389/fpls.2026.1765373)

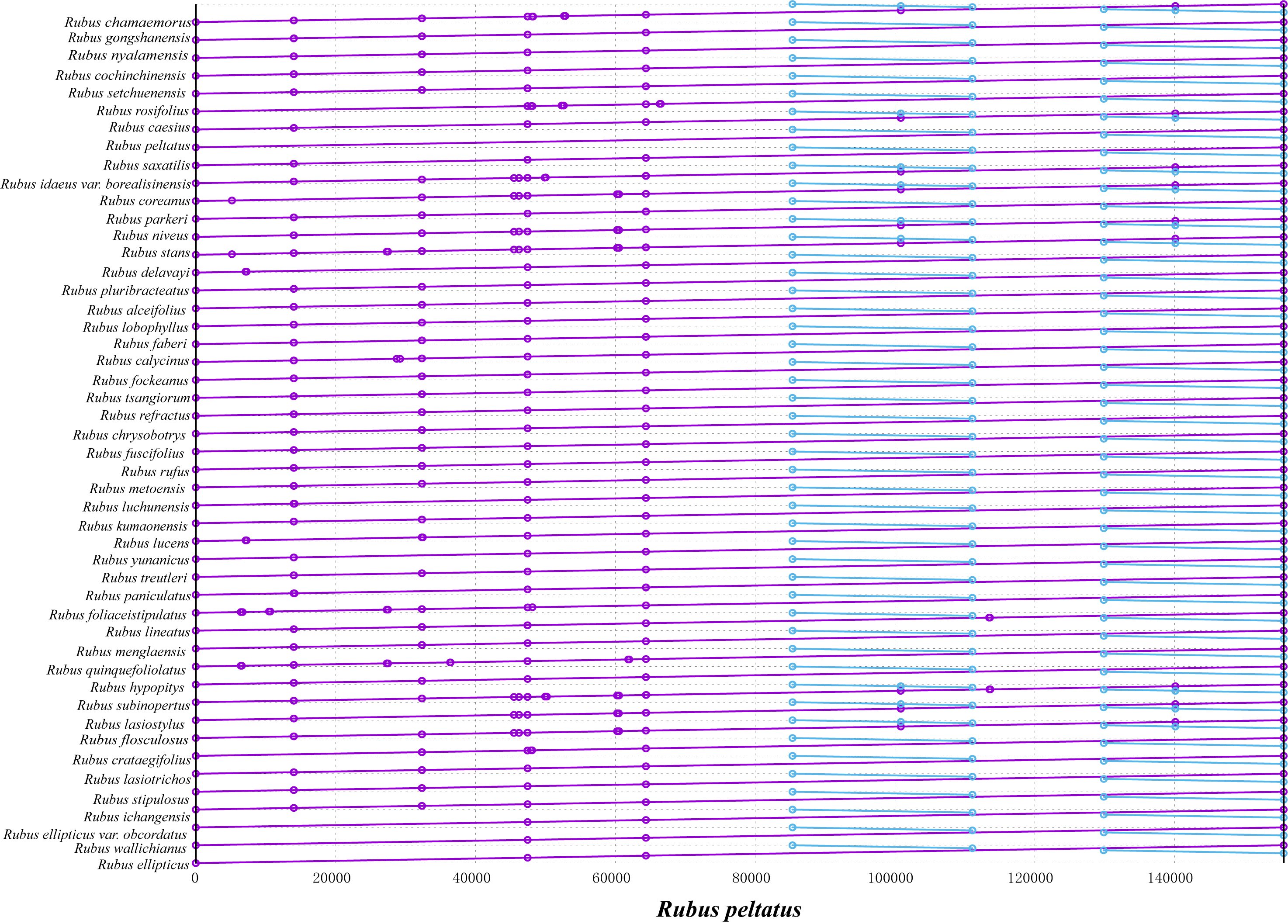

Supplement: Supplementary file 1 [file DataSheet1.zip › 附表20260216/Figure S1 .tif]

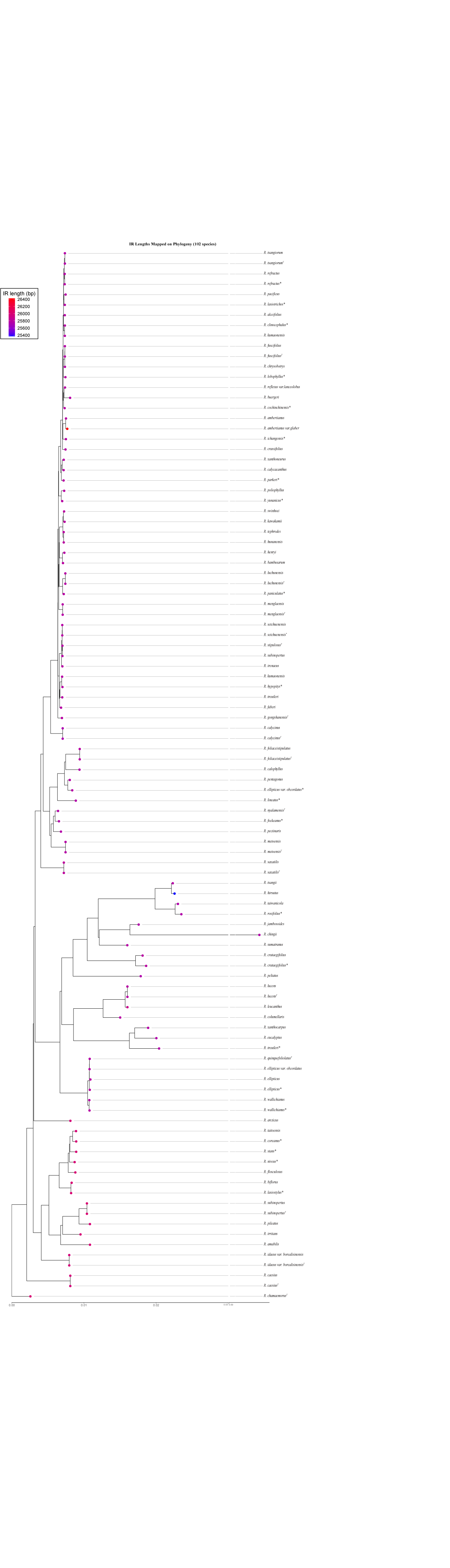

Supplement: Supplementary file 1 [file DataSheet1.zip › 附表20260216/Figure S2 .tif]
